# Estimating the Impact of Earlier ART Initiation and Increased Testing Coverage on HIV Transmission among Men Who Have Sex with Men in Mexico using a Mathematical Model

**DOI:** 10.1371/journal.pone.0136534

**Published:** 2015-08-24

**Authors:** Yanink Caro-Vega, Carlos del Rio, Viviane Dias Lima, Malaquias Lopez-Cervantes, Brenda Crabtree-Ramirez, Sergio Bautista-Arredondo, M. Arantxa Colchero, Juan Sierra-Madero

**Affiliations:** 1 Faculty of Medicine, Universidad Nacional Autónoma de México, Mexico City, Mexico; 2 Department of Infectious Diseases, Instituto Nacional de Ciencias Médicas y Nutrición Salvador Zubiran, Mexico City, Mexico; 3 Hubert Department of Global Health, Rollins School of Public Health & Emory Center for AIDS Research, Atlanta, Georgia, United States of America; 4 Division of AIDS, Department of Medicine, Faculty of Medicine, University of British Columbia, Vancouver, British Columbia, Canada; 5 Division of Health Economics and Health Systems Innovations, National Institute of Public Health, Cuernavaca, Morelos, Mexico; University of New South Wales, AUSTRALIA

## Abstract

**Objective:**

To estimate the impact of late ART initiation on HIV transmission among men who have sex with men (MSM) in Mexico.

**Methods:**

An HIV transmission model was built to estimate the number of infections transmitted by HIV-infected men who have sex with men (MSM-HIV+) MSM-HIV+ in the short and long term. Sexual risk behavior data were estimated from a nationwide study of MSM. CD4^+^ counts at ART initiation from a representative national cohort were used to estimate time since infection. Number of MSM-HIV+ on treatment and suppressed were estimated from surveillance and government reports. Status quo scenario (SQ), and scenarios of early ART initiation and increased HIV testing were modeled.

**Results:**

We estimated 14239 new HIV infections per year from MSM-HIV+ in Mexico. In SQ, MSM take an average 7.4 years since infection to initiate treatment with a median CD4^+^ count of 148 cells/mm^3^(25^th^-75^th^ percentiles 52–266). In SQ, 68% of MSM-HIV+ are not aware of their HIV status and transmit 78% of new infections. Increasing the CD4^+^ count at ART initiation to 350 cells/mm^3^ shortened the time since infection to 2.8 years. Increasing HIV testing to cover 80% of undiagnosed MSM resulted in a reduction of 70% in new infections in 20 years. Initiating ART at 500 cells/mm^3^ and increasing HIV testing the reduction would be of 75% in 20 years.

**Conclusion:**

A substantial number of new HIV infections in Mexico are transmitted by undiagnosed and untreated MSM-HIV+. An aggressive increase in HIV testing coverage and initiating ART at a CD4 count of 500 cells/mm^3^ in this population would significantly benefit individuals and decrease the number of new HIV infections in Mexico.

## Introduction

The HIV epidemic in Mexico is concentrated in men who have sex with men (MSM), among whom the estimated prevalence is 17% [1] compared to 0.24% in the general population [2]. According to national surveillance reports, 54% of AIDS cases in men and 44.3% in the entire population occur in MSM [3]. Women, on the other hand, comprise 17.9% of the total accumulated cases of AIDS in Mexico until 2013 [4]. Most women acquired HIV by sexual contact with their regular male partners [5] who were having concurrent sex with men [2]. In Mexico, there is universal access to Active Antiretroviral Treatment (ART) since 2001, which has produced undeniable benefits to treated people [6]; however, the mortality rate associated with HIV infection has declined less than expected between 2008 and 2011 [2, 3]. In addition to a potential heterogeneity in the quality of the care services, a possible reason for the lack of a larger effect on mortality of such widespread access to ART may be the high frequency of late diagnosis and poor linkage to care, which delay the initiation of ART treatment [7, 8].

ART has been used as a highly effective way to prevent vertical HIV transmission for almost 20 years [9]. More recently, the effect of treating HIV-infected individuals to prevent the sexual transmission of HIV has been demonstrated in cohort studies, ecological studies and clinical trials [10–15]. In addition, modeling studies have estimated substantial reductions in HIV incidence by way of early HIV detection, the scaling-up of treatment and immediate ART initiation strategies [16, 17]. Consequently, for HIV-infected individuals ART is now considered a key component of the combination of strategies available to prevent HIV transmission [18].

The evidence supporting the strategy of “Treatment as Prevention” for HIV assumes a high level of detection among HIV-infected individuals, with widespread ART coverage as well as high linkage to care and retention among those on treatment [18–20]. Thus, a key factor that needs to be considered is the impact of late diagnosis on HIV transmission, given that individuals who seek treatment late very likely spend long periods of time living with uncontrolled viremia and, thus, potentially transmitting their virus to others [7]. A clinical trial designed to study this issue would require a high investment of time and money. Studies based on mathematical modeling offer an alternative to those costly studies, since they provide a tool to compare the impact of different interventions on the desired outcomes [11, 18, 21, 22].

In Mexico, the prevalence of late initiation of ART among HIV-infected individuals ranges between 43 and 79% [7, 23], and only 32% of MSM HIV+ are aware of their status [1]. There are no estimates of the mean time that HIV-infected individuals spend without knowing their HIV status, and of the time they take to enter care and initiate ART from the moment of infection. In addition, the impact that this delay may have on HIV transmission has not been studied, in spite of the high prevalence of late diagnosis and late ART initiation reported, especially among MSM. The aim of this study was to estimate the effect of late diagnosis and late ART initiation on the course of the HIV epidemic among MSM in Mexico from 2011 to 2030.

## Methods

An HIV transmission model based on differential equations was built to estimate the number of infections transmitted by HIV-infected MSM aged 15–54 years who presented late to clinical care after HIV infection. The model ran for 20 simulated years, starting in 2011. A compartmental model divides the population in groups (compartments) and defines the rates of transfer between those compartments, as derivatives of their size with respect to time. This kind of deterministic model assumes that the behavior of a population is completely determined by pre-defined assumptions and does not allow individual variability to account for random distribution of different parameters to reach individual outcomes. In spite of this, we can get mean results that could be easily used in programmatic interventions.

The model compartments represented key stages in the HIV continuum of care: HIV-negative, (HIV-positive) undiagnosed, diagnosed, not on ART, on ART but not virologically suppressed, and on ART and virologically suppressed (an individual under treatment and with a HIV-RNA viral load lower than 400copies/mL is virologically suppressed)—as shown in Fig A in S1 File, in Supporting Information. We estimated monthly transmission using information about sexual risk behavior of MSM, awareness of serostatus and treatment status. Differential equations representing changes along time between compartments of the model are listed in (Eqs 1–5) and detailed information about assumptions and parameters estimation is included into the supporting information. To account for different infectiousness levels, we included three different risk groups: Very High, High and Low risk. These risk groups were selected arbitrarily according to the use of condom in the three last anal sexual relations and to the number of sexual partners per month, both estimated from data from a Mexican seroprevalence study [1]. The ´Very High risk group´ included MSM who reported not using condom in the three last anal sexual relations and having had more than six sexual partners in the last month. The ´Low risk group´ included those who reported always using condom, having no more than one sexual partner in the last month and taking an insertive role in the last three sexual encounters. The ´High risk group´ included persons who could not be classified in any of the other groups (Table C in S1 File). A key assumption in the model was that the individuals in each risk group may have sexual contact with individuals in any of the other groups. We also estimated the number of infections transmitted along time in each scenario using two other definitions for risk groups as a multivariate sensitivity analysis (S1 File, page 11).

$$\frac{\text{dS}}{\text{dt}}=\alpha \;*\text{ N}-\text{S }*\;\left(\lambda_{\text{Inodx}}+\lambda_{\text{Idx}}+\lambda_{\text{Tnosup}}+\lambda_{\text{Tsup}}+\text{m}\right)$$ (1)

$$\frac{{\text{dI}}_{\text{nodx}}}{\text{dt}}=\text{S }*\;\left(\lambda_{\text{Inodx}}+\lambda_{\text{Idx}}+\lambda_{\text{Tnosup}}+\lambda_{\text{Tsup}}\right)-{\text{I}}_{\text{nodx}}\;*\;\left(\rho +\text{m}+\mu \right)$$ (2)

$$\frac{{\text{dI}}_{\text{dx}}}{\text{dt}}=\rho \;*\;{\text{I}}_{\text{nodx}}-{\text{I}}_{\text{dx}}\left(\tau +\text{m}+\mu \right)$$ (3)

$$\frac{{\text{dT}}_{\text{nosup}}}{\text{dt}}=\tau \;*\;{\text{I}}_{\text{dx}}-{\text{T}}_{\text{nosup}}\left(\sigma +\text{m}+\mu \right)$$ (4)

$$\frac{{\text{dT}}_{\text{sup}}}{\text{dt}}=\sigma \;*\;{\text{T}}_{\text{nosup}}-{\text{T}}_{\text{sup}}\left(\text{m}+\mu \right)$$ (5)

**Notes**: ***N*** is the size of the MSM population at the beginning of the simulation; ***S*** is the susceptible population; ***Inodx*** as the infected population unaware of their seropositive status; ***Idx*** is the infected population aware of their seropositive status but not on HAART; ***Tnosup*** is the infected population in treatment not virologically suppressed; ***Tsup i***s the infected population in treatment and virologically suppressed. λ_Inodx_ λ_Idx_ λ_Tnosup_, λ_Tsup_ represent the force of infection for each group of potential transmission. Other parameters are explained in detail in the supporting information.

To validate the model, we compared the number of deaths estimated by the model in a period of 10 years, starting in 2000, with the official reports of the period. The details of the model, the differential equations, parameters and initial conditions are described in the Supplementary material. The code behind the model was built using Berkeley Madonna Version 8.3.18 and the Runge-Kutta 4 integration method; the code is available from the authors.

We explored five intervention scenarios defined according to the median CD4^+^ count at ART initiation and the percentage of undiagnosed HIV-positive MSM. The scenarios were constructed based on the more likely hypothetic conditions derived from worldwide and local recommendations, as well as a significant increase in diagnosis coverage reaching up to 80% of affected individuals. These scenarios were:

- *Status quo*: current conditions with a median CD4^+^ count at ART initiation of 148cells/mm^3^ (25^th^-75^th^ percentiles: 52–266) and 32% of individuals aware of their infection;
- ART initiation according to Mexican guidelines: CD4+ count of 350cells/mm^3^ and 32% of individuals aware of their infection;
- ART initiation according to WHO guidelines: CD4^+^ count of 500cells/mm^3^ and 32% of individuals aware of their infection;
- ART initiation according to Mexican Guidelines: CD4^+^ count of 350 cells/mm^3^ and 80% of individuals aware of their status;
- ART initiation according to WHO Guidelines: CD4^+^ count of 500 cells/mm^2^ and 80% of individuals aware of their status.

CD4^+^ count at diagnosis and at ART initiation were estimated from the MSM population in a Mexican cohort of HIV positive individuals followed at the Instituto Nacional de Ciencias Médicas y Nutrición Salvador Zubirán (INCMNSZ) in Mexico City. Information from patients was anonymized and de-identified prior to analysis; their data are available upon request to the authors. The characteristics of this cohort have been described in previous publications [6, 7] and the studies using this cohort have been approved by the *Comite de etica en Investigacion*. *Registered in the Federalwide Assurance (FWA) for the Protection of Human Subjects (Number*: *FWA00014416)*. We used CD4^+^ count at diagnosis and at ART initiation to assess the time that MSM spent without treatment since becoming infected, extrapolating the results estimated by a published study [24] that showed a decline of CD4^+^ counts in a cohort of recently seroconverted MSM individuals. We described the details of the estimation in the Supplementary material. Sexual risk behavior (which was used to define the different risk groups), HIV prevalence and the percentage of individuals aware of their infection, were estimated from a recent nationwide seroprevalence study [1]. Information in this database is anonymous and the identity of the participants in the study could not be stated, their data are available upon request to the researchers. The number of HIV infections in the country in 2011 was estimated from a UNAIDS report [25]. The remaining parameters, such as mortality rates, patients on treatment and the proportion of virologically suppressed individuals were estimated from Mexican official reports [2, 26]. Data from official reports are publicly available.

Univariate sensitivity analyses were performed on the following parameters: percentage of condom use, number of sexual partners per month in each risk group, number of sexual contacts per sexual partner per month, rate of HIV transmission by sexual role (insertive/receptive) and rate of viral suppression of individuals on ART treatment.

## Results

In the *Status Quo* scenario, HIV-infected MSM spent an average of 7.4 years after infection before initiating ART, which occurred when the median CD4^+^ T-cell count was 148 cells/mm^3^(25^th^-75^th^ percentiles: 52–266). In this scenario, 68% of MSM living with HIV were not aware of their HIV status and transmitted 78% of the total estimated new infections in the first year of simulation. The total number of infections transmitted to other MSM in the first year of the *Status Quo* scenario is 14239. Of these infections, 32 originated from treated and virologically suppressed MSM, 1421 from treated but not virologically suppressed MSM, 1587 from MSMS diagnosed with HIV+ but not treated and 11198 from undiagnosed HIV-infected MSM. In the second scenario (ART initiation according to Mexican guidelines at CD4^+^ count of 350cells/mm^3^), the time from infection to ART initiation was estimated at 2.8 years. In this scenario, the total number of transmitted infections in the first year was 14159. The third scenario (ART initiation according to WHO guidelines at CD4^+^ count of 500cells/mm^3^) implied an average time of 0.67 years since infection and resulted in 14013 transmitted infections. If the detection of HIV increased to 80% of HIV-infected MSMs, the number of transmitted infections in the first year would be 13933 and 13546 for a CD4^+^ count at ART initiation of 350 and 500cells/mm^3^, respectively (Table 1). In supporting material, the number of transmitted infections along time by group for the scenario “HAART initiation according to WHO guidelines + 80% of diagnoses “is shown in Table F in S1 File.

**Table 1:** Estimation of number of infections transmitted in the first year of simulation by HIV awareness and treatment status in different scenarios modeled.

Scenario Simulated					
HIV awareness and treatment status	Status Quo	HAART initiation according to Mexican guidelines	HAART initiation according to WHO guidelines	HAART initiation according to Mexican guidelines + 80% of diagnoses increased	HAART initiation according to WHO guidelines + 80% of diagnoses increased
**HIV+ not diagnosed**	11,198	11,196	11,192	7,278	7,269
**HIV+ diagnosed**	1,587	1,088	350	3,309	1,143
**Treated not suppressed**	1,421	1,842	2,437	3,312	5,096
**Treated and suppressed**	32	33	34	35	38
**Total MSM**	**14,239**	**14,519**	**14,013**	**13,933**	**13,546**

**Fig 1:**
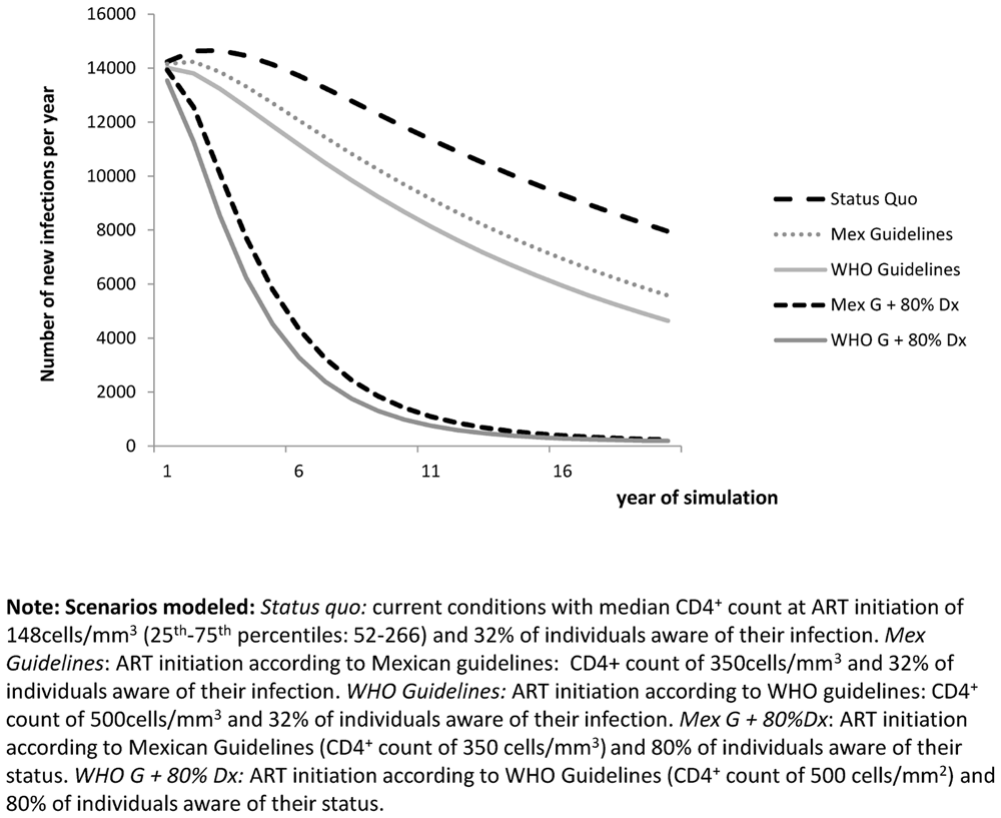
Estimated number of new infections over time in each simulated scenario.

Even though the decrease in transmission cases was small in all scenarios during the first year compared to the *Status Quo*, a more important decrease was observed when the projected periods of time were longer. Fig 1 shows the trend of transmitted HIV infections, projected in the 5 scenarios up to 20 years into the future. In these projections, the scenarios with early ART initiation but no increase in the proportion of MSM diagnosed individuals resulted in a decrease of transmitted HIV infections of 0.5% in the first year, 5.3% in the fifth year, 9.9% in the tenth year and 16% in the 20th year if ART initiation ocurred according to Mexican guidelines. If ART was initiated according to WHO guidelines, transmitted HIV infections would decrease by 1.6% in the first year, 9.3% in the fifth year, 15.6% in the tenth year and 23.5% in the 20th year. In both scenarios, increasing the proportion of diagnosed HIV-infected MSM to 80% of new infections resulted in a decrease of transmitted HIV infections of 2.1%, 30.6%, 53.4% and 70.4% at 1, 5, 10 and 20 years of simulation, respectively, when ART was initiated according to Mexican guidelines; and 4.9%, 38.8%, 60% and 75% when ART was initiated according WHO guidelines. There was a considerably more significant decrease in transmission in these contexts compared to the other scenarios. The accumulated number of infections transmitted by MSM in each scenario over the 20 year projection and the percentage of reduction relative to Status Quo scenario for hypothetical scenarios are shown in Table 2.

**Table 2:** Number of accumulated infections and relative reductions compared to the Status Quo in each scenario simulated along time.

	Scenario simulated				
Year	Status Quo N	HAART initiation according to Mexican guidelines N (% reduction)	HAART initiation according to WHO guidelines N (% reduction)	HAART initiation according to Mexican guidelines + 80% of diagnoses increased N (% reduction)	HAART initiation according to WHO guidelines + 80% of diagnoses increased N (% reduction)
**1** ^**st**^ **year**	14,239	14,159 (0.6)	14,013 (1.6)	13,933 (2.1)	13,546 (4.9)
**5** ^**th**^ **year**	72,134	68,271(5.4)	65,448 (9.3)	50,088 (30.6)	44,108 (38.8)
**10** ^**th**^ **year**	136,002	122,512 (9.9)	114,838 (15.6)	63,356 (53.4)	53,811 (60.4)
**15** ^**th**^ **year**	188,526	163,589 (13.2)	150,818 (20)	67,015 (64.4)	56,347 (70.1)
**20** ^**th**^ **year**	231,599	194,776 (15.9)	177,141 (23.5)	68,530 (70.4)	57,491 (75.2)

N = number of acummulated infections. Percentage of reduction relative to Status Quo scenario.

**Fig 2:**
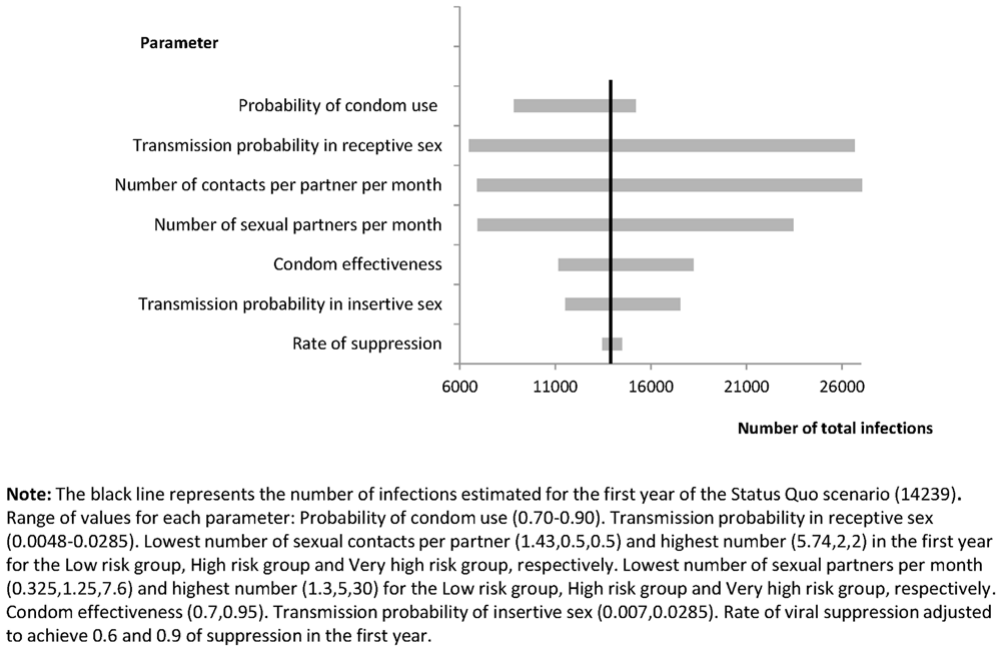
Number of infections estimated for the first year of the simulated Status Quo scenario due to changes in the selected parameters.

**Fig 3:**
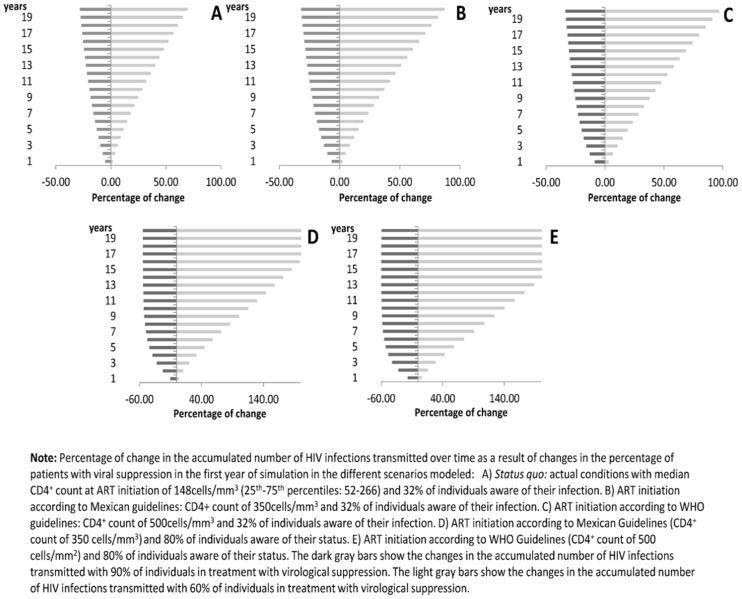
Predicted percent change in the accumulated number of infections due to hypothetic changes in the rate of virological suppression (60% or 90%) in each scenario modeled over time.

A univariate sensitivity analysis was performed in the *Status Quo* scenario to determine the influence on the estimated number of infections of possible changes in the parameters considered [27]. Results are shown in Figs 2 and 3. According to this analysis, if the proportion of condom use increased from 73% to 90%, the number of new transmitted infections would decrease by 38% during the first year. If the efficacy of condoms to prevent HIV transmission were as low as 70%, the number of new infections would increase by 28% in the first year. The probability of transmission through receptive anal sex had the most important influence in the results, since the number of infections increased by 80% when the probability was increased to 2.85%. However, an increase to 90% in the percentage of individuals with suppressed viral load in the first year of simulation would translate into a reduction of only 5% in the number of infections transmitted in the first year, with small changes over time, reaching a 28% decrease in transmissions in the 20^th^ year (Panel A in the Fig 3). The effect over time of having 90% of individuals on treatment virologically suppressed under the different scenarios is shown in Fig 3. Because the definition of risk groups in our model was arbitrary, a sensitivity analysis changing the definition of risk group was made and the results on transmitted infections over time for each definition and scenario were calculated (Table G in S1 File). Although the results were different from the main analysis, the scenarios with higher diagnostic coverage maintain the effect on reduced transmission as the original analysis showed, with reductions in transmission of up to 86% in comparison with the Status Quo in the twentieth year of simulation.

## Discussion

Using mathematical modeling based on data obtained from different sources in Mexico, this study aimed to project the number of HIV infections transmitted by the MSM population, in different hypothetical scenarios compared to the Status Quo scenario. The Status Quo scenario is the one of ART initiation at late stages of disease and a high proportion of undiagnosed individuals living with HIV in Mexico. Our data showed that initiating ART treatment at earlier stages had a significant impact on HIV transmission when following the current recommendations of the WHO or even the more conservative Mexican Guidelines of 2013. More importantly, the model showed that undiagnosed (and therefore untreated) HIV-infected individuals were the major drivers of transmission and they should be targeted by different prevention strategies implemented in Mexico.

Under the current conditions in which ART is initiated in late stages of the disease and there is a high proportion of undiagnosed individuals with HIV among MSM (68%), we estimated that there would be 231599 new transmitted infections over the next 20 years that could be potentially averted. A strategy of initiating antiretroviral treatment earlier, according to current Mexican or WHO guidelines, would produce a moderate reduction in transmitted infections. However, if HIV testing was substantially increased among MSM subjects until it covered at least 80% of infected individuals (assuming a high percentage of linkage to care), the reduction in transmitted infections would be very high.

The crucial role played by undiagnosed HIV-infected individuals in sustaining transmission in a given region was recently recognized by Eyawo et al. [28] and it was clearly confirmed by our results. Our model was validated by the number of new cases projected in MSM individuals in the first year under the *Status Quo* scenario, which was approximately 16% lower than that reported by the Mexican Health Ministry [4] for the year 2012, accounting for 68% of new undiagnosed infections.

The effect of initiating ART early and testing most of the HIV-infected individuals unaware of their diagnosis has been shown in models published by others [22, 29, 30]. Our model is unique in showing with local data how a self-sustained HIV epidemic in our country can be curtailed in a medium and long term period by implementing aggressive testing and treatment strategies. The implementation of these strategies poses significant challenges. For instance, reducing the high rate of late ART initiation in a given population entails a comprehensive understanding of the factors involved, which may include social, educational and psychological barriers as well as factors related to access and retention in care. Campaigns to encourage HIV testing among high risk groups in the last years have resulted in a doubling of the number of tests and detections [31] between 2006 and 2011, according to official reports [32]. However, in a recent study performed in Mexico by Bautista *et al*. (with approximately 8000 MSM), a large proportion of HIV-infected MSM were unaware of their HIV status [1], and only 45% had been tested for HIV at least once in their lifetime; moreover, of those found HIV positive (close to 20%), 68% were not aware of their seropositive status [1]. This clearly highlights that HIV testing campaigns in Mexico does not reach the population that is the main driver of the Mexican HIV epidemic in a timely and effective manner. Regulatory barriers associated with HIV testing, such as the still mandatory written informed consent, may hinder the increase of the rate of HIV testing. In addition, the stigma and discrimination against homosexuality, sex work and HIV infection may also play an important role [33, 34]. Nevertheless, few intervention studies have assessed the effect of reducing stigma to improve HIV testing in the country [35]. Denial among MSM may be another factor; this issue was addressed in a recent survey study in Mexico City [36], in which 13% of men attending meeting and gathering sites of the MSM population refused to participate saying they were heterosexual [36].

Our model has some limitations. First, a significant proportion of transmissions may originate from individuals in the acute phase of the infection, probably due to the higher level of HIV viral load in plasma and body fluids [37, 38]. Our model assumed a constant transmissibility during all the time that the infection remains untreated, and may thus underestimate the number of transmissions during the acute phase; the potentially preventable infections could thus be higher. While we recognize that CD4 at HAART initiation in patients with chronic infection may not be a totally accurate marker for time of infection, it is the best marker we have for our model and the information is based on cohort studies such as Cascade and PRIMO[24] in which the time of seroconversion is known.

Second, our model assumed that HIV care of all Mexican clinical providers, specifically the ART prescription and the monitoring of the patients, is homogeneous in quality. Given that we used a mean viral suppression rate as a measure of clinical success, which could overestimate the real effectiveness of ART, the benefit on preventable infections could be lower. However, we estimated that the impact of individuals treated but not suppressed is important along time and we think that additional interventions to increase viral load suppression would still have a high preventive potential if they were implemented and focused on this group. Another limitation is that the effect of the stage of HIV on sexual behavior was not considered in the model. Individuals with symptomatic HIV infection in advanced stages of the disease may actually pose less risk of transmitting their HIV infection, while individuals under treatment who feel well and increase their sexual risk behavior could increase their risk of transmission. The model represents the mean behavior of individuals and, therefore, its results were an estimate (albeit a fair one) of the mean number of HIV transmissions in the Mexican population. The model predicts events occurring as far as 20 years from baseline, using parameters collected in current time which may not necessarily occur farther, and therefore may incorrectly estimate the rate of transmissions in the later years. Finally, due to the lack of data we did not explore scenarios with HIV transmission in women, who, according to previous results, were infected by men in 87% of the cases [2]. However, an intervention to reduce HIV transmission in MSMs would also lead to a decrease of infections in the population of Mexican women.

In summary, according to our results, a significant number of new HIV infections in Mexico were transmitted by undiagnosed and untreated MSM-HIV+. This result highlights the urgent need for health policies that aggressively increase the coverage of HIV testing in the MSM population; such a strategy would probably produce significant benefits for individuals and for the efforts to control the HIV epidemic in the country in the short and long term. Further studies should aim to estimate the cost of implementing these interventions and support our recommendations.

## Supporting Information

S1 File Fig A, Scheme of the transmission model. Fig B, Polynomial fit for CD4 count decline used to estimate the time since infection to different CD4 counts. Table A, List of parameters and initial conditions of the transmission model. Table B, Parameters used in the estimation of force of infection. Table C, Percentage of MSM by risk group, prevalence of HIV and mean number of sexual partners in the last month. Table D, Number of deaths in HIV-infected MSM estimated by the model in comparison with the official number reported. Table E, Estimation of time since infection fitted to CD4 counts at diagnoses and HAART initiation. Table F, Estimation of the number of accumulated infections transmitted along time by HIV awareness and treatment status in the scenario of HAART initiation according to WHO guidelines + 80% of diagnoses. Table G, Number of accumulated infections transmitted in each scenario simulated along time using different definitions of risks groups. (DOCX)
