# Supplementary material for: Estimating the Impact of Earlier ART Initiation and Increased Testing Coverage on HIV Transmission among Men Who Have Sex with Men in Mexico using a Mathematical Model
Source: PLoS One. 2015 Aug 24;10(8):e0136534. doi: 10.1371/journal.pone.0136534 (PMC4547810; doi:10.1371/journal.pone.0136534)
Supplement: S1 File — (DOCX) [file pone.0136534.s001.docx]

**Supporting Information**

***Description of the model***

We proposed a compartmental model to show the evolution of the HIV epidemic in the MSM population in Mexico. We defined ***N*** as the size of the MSM population at the beginning of the simulation; ***S*** as the susceptible population (i.e., individuals at risk of acquiring HIV); ***Inodx*** as the infected population unaware of their seropositive status; ***Idx*** as the infected population aware of their seropositive status but not on HAART; ***Tnosup*** as the infected population in treatment not virologically suppressed; ***Tsup*** as the infected population in treatment and virologically suppressed. HIV infected MSM were classified by sexual behavior as Very High, High and Low risk. The parameters used to estimate transition rates between stages and the number of individuals in each compartment at the beginning of the simulation (Initial conditions of the model) are listed in Table A. A scheme of the model is shown in **Figure A** and the set of differential equations are listed below.

**Figure A**. Scheme of the transmission model


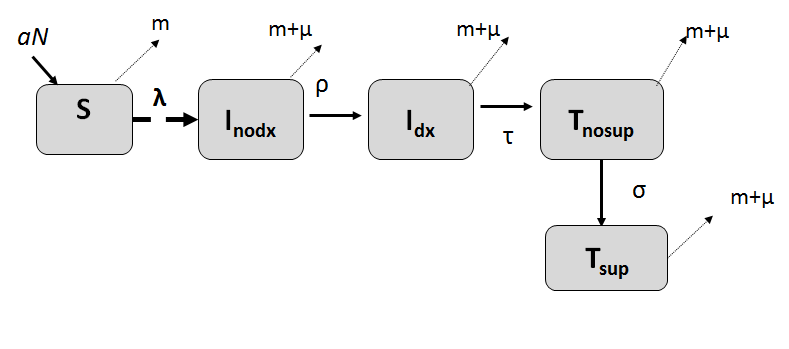


The differential equations are described by:

$$\frac{\mathrm{dS}}{\mathrm{dt}}=\alpha*N-S*\left( \lambda_{\mathrm{Inodx}}+\lambda_{\mathrm{Idx}}+\lambda_{\mathrm{Tnosup}}+\lambda_{\mathrm{Tsup}}+m \right)$$

$$\frac{\mathrm{dI}_{\mathrm{nodx}}}{\mathrm{dt}}=S*\left( \lambda_{\mathrm{Inodx}}+\lambda_{\mathrm{Idx}}+\lambda_{\mathrm{Tnosup}}+\lambda_{\mathrm{Tsup}} \right)-I_{\mathrm{nodx}}*\left( \rho+m+\mu\right)$$

$$\frac{\mathrm{dI}_{\mathrm{dx}}}{\mathrm{dt}}=\rho*I_{\mathrm{nodx}}-I_{\mathrm{dx}}\left( \tau+m+\mu\right)$$

$$\frac{\mathrm{dT}_{\mathrm{nosup}}}{\mathrm{dt}}=\tau*I_{\mathrm{dx}}-T_{\mathrm{nosup}}\left( \sigma+m+\mu\right)$$

$$\frac{\mathrm{dT}_{\sup}}{\mathrm{dt}}=\sigma*T_{\mathrm{nosup}}-T_{\sup}(m+\mu)$$

Our key assumptions were:

- An individual who starts HAART and whose viral load reaches an undetectable level will stay undetectable as long as the treatment is not interrupted.
- The viral suppression level reported was based on Mexican official data, considering those who interrupted the treatment during follow up as not suppressed.
- The probability of transmission by sexual contact between an infected and a susceptible person depends on risk behavior (number of sexual contacts per partner, number of sexual partners per month, condom use and type of sexual practice: anal insertive or receptive), adhesion to treatment by infected men and their viral suppression status.
- The sexual risk behavior of each individual is independent of its treatment and viral suppression status.
- The CD4 count at the moment of infection and posterior decline are similar to what was reported for MSM individuals included in the CASCADE cohort [[1](#_ENREF_1)].

**Table A. List of parameters and initial conditions of the transmission model**

| **Parameter** | **Description** | **Point estimate**  **(Range)** | **Source** |
| --- | --- | --- | --- |
| α | Monthly recruitment rate into the susceptible population | 0.0411 | The median age of sexual debut in MSM is 17 years [[2](#_ENREF_2)]. |
| N_0_ | Total number of the population of men between 15 to 54 years at the end of 2011 | 30,960,084 | Estimate based on the number of men alive (Information available for 2010 only [[3](#_ENREF_3)]) |
| S_0_ | Number of susceptible MSM | 1,688,600 | Assuming 6% of men are MSM [[4](#_ENREF_4)]. |
| $\lambda_{\mathrm{Inodx}}\lambda_{\mathrm{Idx}}$ $\lambda_{\mathrm{Tnosup}},\lambda_{\mathrm{Tsup}}$ | Force of infection for each group of potential transmission | Estimated each month in the software | See the section: Estimation of force of infection. |
| m | Monthly mortality rate due to other causes | 0.00029 | Background mortality rate for men between 15 and 54 years of age [[5](#_ENREF_5)]. |
| Inodx_0_ | Number of HIV-infected MSM who are not aware they are infected | 114,924 | 68% of infected people are not aware of their status [[2](#_ENREF_2)]. |
| ρ | Monthly rate of HIV-positive diagnoses in the actual and increased diagnostic scenarios | 0.015  0.09 | 1/Time (in months) since infection to HIV-positive diagnosis with a CD4 of 209 cells/mm^3^, the median CD4 at diagnosis estimated in the MSM cohort (See Table E) and polynomic fit for the scenario of 32% of diagnosis, or 80% of diagnosis respectively |
| µ | Monthly mortality rate in HIV individuals (Oficial reports of deaths by HIV are not necessarily attributed to AIDS) | 0.0027 | Of an estimate of 97,473 MSM-HIV+ in 2011, 3,189 died [[6](#_ENREF_6)]. |
| Idx_0_ | Number of HIV-infected and diagnosed MSM | 54,082 | 82% of eligible males (diagnosed) are under treatment [[2](#_ENREF_2)]. |
| τ | Monthly rate of treatment initiation in the *Status quo scenario , the* ART initiation according to Mexican guidelines and ART initiation according to WHO guidelines scenarios respectively. | 0.054  0.1439  0.6015 | 1/Time (in months) between a positive diagnosis and treatment initiation. Estimated from cohort information ( Table E). |
| T_nosup0_ | Number of treated and non-suppressed MSM | 12,861 | 70,219 individuals older than 15 years are in treatment and 78% of them are males [[7](#_ENREF_7)]; thus, 44,347 MSM are treated and 71% of them are virologically suppressed [[8](#_ENREF_8)]. |
| σ | Monthly viral load suppression rate | 0.04 | Estimated to reach 71% of treated individuals with viral load suppression (VL<400) with at least 6 months on ART [[8](#_ENREF_8)]. |
| Tsup_0_ | Number of MSM on HAART who achieved viral suppression | 31,486 | 71% of individuals under treatment are suppressed [[9](#_ENREF_9)]. |

- *Note: The subscript zero refers to the number under the initial conditions. The total number, according to official reports, of MSM HIV+ is 105,984 (54,082 diagnosed, 44,347 under treatment and 31,486 of of them under suppression). According to our estimations, the real number would be 169,005 MSM, distributed as: 114,924 undiagnosed (68% of HIV+), 54,082 diagnosed, and 44,347 under treatment, 31,486 of which are suppressed).*

**Table B. Parameters used in the estimation of force of infection.**

| **Parameter** | **Description** | **Estimation and Value** |
| --- | --- | --- |
| $p$ | Number of sexual partners of a susceptible MSM per month | Weighted by each risk group. 0.65, 2.5 and 15.2 partners for the low, highand very high risk groups, respectively. Estimated based on the database of a Mexican seroprevalence study [[2](#_ENREF_2)]. |
| $n$ | Number of sexual contacts per partner per month | Based on the database of a Mexican seroprevalence study [[2](#_ENREF_2)] and estimated at 2.87, 1 and 1 sexual contacts per partner in Low, High and Very High risk groups, respectively. |
| $c$ | Proportion of sexual acts with condom use | 0.73 (0.71-0.74) [[2](#_ENREF_2)]. |
| $e$ | Condom efficacy | 0.84 of individuals who always used a condom did not report problems such as breakage, slippage or delayed application [[10](#_ENREF_10)]. |
| $r$ | Probability of being receptive | 0.64, estimated from the database of the Mexican seroprevalence study. Includes individuals reporting both insertive and receptive roles [[2](#_ENREF_2)]. |
| $\rho$ | Transmission probability of HIV in a receptive sexual contact | 0.0143 (0.0048-0.0285)[[11](#_ENREF_11)]. |
| $1-r$ | Probability of being insertive | 0.36 of the individuals reported only insertive anal sex in the last sexual encounter. From database of Mexican seroprevalence study [[2](#_ENREF_2)]. |
| $\iota$ | Transmission probability for insertive sex | 0.0062(0.007-0.0168) [[11](#_ENREF_11)]. |

*Sexual risk Groups:*

We arbitrarily defined three sexual risk groups in order to keep the differences of HIV prevalence observed in the database of the Mexican seroprevalence study [[2](#_ENREF_2)]. The groups were defined as follows: The ´Very High risk group´ included MSM who reported not using condom in the three last anal sexual relations and having had more than six sexual partners in the last month. The ´Low risk group´ included those who reported always using condom, having no more than one sexual partner in the last month and taking an insertive role in the last three sexual encounters. The ´High risk group´ included people who could not be classified in any of the other groups (Table C).

**Table C. Percentage of MSM by risk group, prevalence of HIV and mean number of sexual partners in the last month.**

| **Risk group** | **% of MSM** | **Prevalence of HIV** | **Mean number of sexual partners**  **(SD)** |
| --- | --- | --- | --- |
| **Very high** | 0.006 | 0.28 | 15.2 (19.7) |
| **High** | 0.064 | 0.17 | 2.5 (5.8) |
| **Low** | 0.93 | 0.12 | 0.65 (0.47) |

*Note: We estimated these data from Bautista study´s Database [*[*2*](#_ENREF_2)*].*

*Force of infection:*

The monthly force of infection rate was estimated for each compartment: diagnosed, undiagnosed, treated unsuppressed and treated suppressed. The expression used to estimate the force of infection is shown in equation (1) and the parameters used in this calculation are presented in Table B.

$$\lambda_{G} =\theta_{G} * \beta_{p} \left( 1 \right)$$

where:

- $\theta_{G}$ is HIV prevalence for each compartment *G* ( undiagnosed, diagnosed, treated unsuppressed and treated suppressed).
- $\beta_{p}$ is transmission probability per sexual partner, weighted by sexual risk group (s = Very High risk, High risk group and Low risk group). We used a Binomial function, as shown in equation (2), where $n_{s}$ is the number of sexual contacts per partner in each sexual risk group, $p_{s}$ is the number of sexual partners per month in each risk group and $\beta_{a}$ is the transmission probability per sexual contact, estimated in equation (3).

$$\beta_{p} = \sum_{s} p_{s}*{[1 - \left( 1 - \beta_{a} \right)^{n_{s}}]}^{p_{s}} (2)$$

$$\beta_{a} = (1-c*e)*[r*\rho+ (1 - r)*\iota] (3)$$

We assumed that, the probability of transmission from suppressed individuals in MSM sero-discordant couples, even if close to zero, cannot be stated in absolute terms as such, and more accurately this probability should be expressed as a range around a confidence interval, as presented in the Partners study (CROI 2014) [17]. Therefore, the value of 0.00008 used in our study, represents the monthly rate of transmission from treated individuals with viral suppression, and was derived from the study of Attia et al [18], in which it was estimated one transmission per 100000 sexual acts, in suppressed individuals. Assuming 100 contacts per year we calculated a constant monthly rate of transmissions per person of 0.00008.The system of differential equations was solved using Berkeley Madonna Version 8.3.18 and the Runge-Kutta 4 integration method for 20 simulated years starting on 2011. Our main result is the number of susceptible MSM individuals who become infected with HIV each month. To validate the model, we ran it with available data for the year 2000 and estimated the mortality in HIV-infected MSMs for the next ten years. Official reports estimated 30,830 deaths in HIV-infected males aged 15-64 years between 2003 to 2010 [[12](#_ENREF_12)]. We assumed that 80% were MSM according to the frequency observed in the INCMNSZ cohort; thus, 24,464 deaths were of MSM. In comparison, the estimate of our model for this period was 30,691 deaths which, assuming the same characteristics for the progression between compartments that we used for the year 2011 and a possible expected underestimation of 25% in the official reports, allow us to consider the results as consistent. The results of the mortality estimates by year are shown in Table D.

**Table D. Number of deaths in HIV-infected MSM estimated by the model in comparison with the official number reported.**

|  | **Number of deaths** | |
| --- | --- | --- |
| **Year** | **Estimated** | **Reported** |
| 2003 | 3708 | 3669 |
| 2004 | 3777 | 3765 |
| 2005 | 3827 | 3708 |
| 2006 | 3860 | 3925 |
| 2007 | 3880 | 3975 |
| 2008 | 3887 | 4076 |
| 2009 | 3883 | 3928 |
| 2010 | 3869 | 3784 |

***Note:*** *We assumed these initial conditions for the year 2000: N_0_=25,557,395 [*[*13*](#_ENREF_13)*]; S_0_=1,432,171 [*[*4*](#_ENREF_4)*], Inodx_0_= 62,032 [*[*14*](#_ENREF_14)*]; Idx_0_= 29,191 [*[*14*](#_ENREF_14)*]; Tsup_0_=7,136 [*[*15*](#_ENREF_15)*]; Tnosup_0_=2,914 [*[*15*](#_ENREF_15)*]*

***Polynomial fitting for time from infection***

A decline of CD4 counts in recent seroconverters was shown in a study of the CASCADE cohort [[1](#_ENREF_1)]. We fitted a polynomial function to the data shown in that study to predict the time elapsed since infection to HIV diagnosis for the CASCADE cohort. We used data of CD4 counts at diagnosis and at HAART initiation for a Mexican MSM-HIV cohort, as described in Table E. We assumed that the patients in this cohort had the same CD4 count at the time of infection as the patients in the CASCADE cohort and the same slope of decline over time. We used the polynomial fit to estimate the time that elapsed in our cohort between the moment of infection to a given CD4 count at diagnosis and at HAART initiation in all modeled scenarios. The results are shown in Table E.

**Figure B.** Polynomial fit for CD4 count decline used to estimate the time since infection to different CD4 counts.


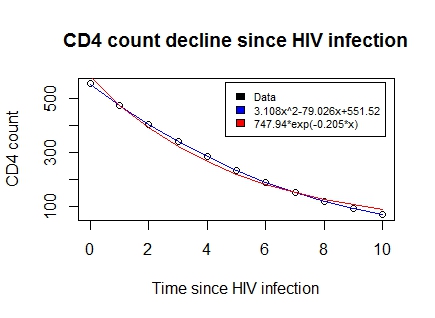


**Table E. Estimation of time since infection fitted to CD4 counts at diagnoses and HAART initiation.**

| **Stage of clinical care** | **Median CD4 count**  **(25th-75th percentile)** | **Estimated time in years** |
| --- | --- | --- |
| At HIV diagnosis | 209 (69 - 371) | 3.43 (1.33 - 6.53) |
| At ART initiation | 148 (52- 266) | 7.07 (4.36 – 11.76) |
| At ideal ART initiation with CD4 cell count of 350 cells/mm^3^ | 350 | 2.87 |
| At ideal ART initiation with CD4 cell count of 500 cells/mm^3^ | 500 | 0.67 |

***Sensitivity Analysis***

Univariate sensitivity analyses were performed on the following parameters: percentage of condom use, number of sexual partners per month per risk group, number of sexual contacts per sexual partner per month, rate of HIV transmission by sexual role (insertive/receptive) and rate of viral suppression of individuals on ART. The range of values within the parameters varied comes from the literature, when available, or from our estimations ( B and C Tables).

We noticed an important percentage of transmission were due to individuals on treatment but not suppressed in Table 1 (manuscript), especially on the scenario “HAART initiation according to WHO guidelines + 80% of diagnoses”. We checked the impact of this group along time in Table F. Additionally, a specific analysis of the rate of suppression over time was developed for each modeled scenario (Figure 3 in the manuscript). We fitted the rate of suppression to 60 and 90% of individuals under treatment in the first year of simulation. We assumed that the rate of suppression would increase over the twenty years of simulation following the same trend of the estimated suppression rate at baseline.

**Table F. Estimation of the number of accumulated infections transmitted along time by HIV awareness and treatment status in the scenario of HAART initiation according to WHO guidelines + 80% of diagnoses.**

| **HAART initiation according to WHO guidelines + 80% of diagnoses** | | | | | |
| --- | --- | --- | --- | --- | --- |
| **HIV awareness and treatment status** | **1^st^ year** | **5^th^ year** | **10^th^ year** | **15^th^ year** | **20^th^ year** |
| **HIV+ not diagnosed** | 7,269 | 13,760 | 15,051 | 15,355 | 15,475 |
| **HIV+ diagnosed** | 1,143 | 2,205 | 2,406 | 2,453 | 2,471 |
| **Treated not suppressed** | 5,096 | 27,686 | 35,157 | 36,650 | 37,060 |
| **Treated and suppressed** | 38 | 457 | 1,197 | 1,889 | 2,485 |
| **Total MSM** | **13,546** | **44,108** | **53,811** | **56,347** | **57,491** |

As our definition for risk groups is not a standard definition but an arbitrary one, we decided to explore the results for two additional definitions for Risk groups. In the first definition we established only two risk groups, according with the use of condom in the last three sexual anal encounters. A low risk group reported using the condom in all the three encounters. This group includes 37.3% of the total population, has a mean of 2.7 sexual partners in the last month, one sexual contact per partner and a sero-prevalence of HIV of 19.5%. On the other hand, the high risk group defined as those who reported not using a condom in at least one of the last 3 sexual encounters, corresponded to 62.7% of the total population. In this group there was a mean of 2.10 sexual partners per month, 2.5 sexual contacts per partner and an HIV prevalence of 15.8%. For the second definition, we established three risk groups: A very high risk group included people with more than 9 sexual partners per month and reporting not using a condom in the last 3 encounters. A low risk group, included people with one sexual partner and reporting always using a condom, and the medium risk group those who do not fit in any of the other groups. With this definition, the proportion in each group were 1%,70% and 29% in very high, medium and low risk respectively and the mean number of sexual partners per month in each group was 22.7, 1.77 and 0.66 in the same order. Using these new definitions, the number of infections transmitted in the first, fifth, tenth and twentieth year in each scenario are shown in the Table G.

**Table G. Number of accumulated infections transmitted in each scenario simulated along time using different definitions of risks groups.**

| **Definition 1:** Two risk groups. Low risk group including people who reported using the condom in all the three encounters. High risk group with those who reported not using a condom in at least one of the last 3 sexual encounters | | | | | |
| --- | --- | --- | --- | --- | --- |
| **Year** | **Status Quo** | **HAART initiation according to Mexican guidelines** | **HAART initiation according to WHO guidelines** | **HAART initiation according to Mexican guidelines + 80% of diagnoses increased** | **HAART initiation according to WHO guidelines + 80% of diagnoses increased** |
| **1^st^ year** | 24,756 | 24,619 | 24,366 | 24,226 | 23,549 |
| **5^th^ year** | 146,958 | 138,921 | 132,823 | 100,596 | 87,447 |
| **10^th^ year** | 344,927 | 306,924 | 284,292 | 143,867 | 117,301 |
| **15^th^ year** | 600,601 | 504,416 | 453,420 | 161,636 | 127,601 |
| **20^th^ year** | 930,216 | 736,541 | 642,332 | 169,665 | 131,847 |
| **Definition 2.** Three risk groups. A high risk group including people with more than 9 sexual partners per month and reporting never use a condom. A low risk group, including people with one sexual partner and reporting always using a condom in the last three sexual anal encounters. The medium risk group those who do not fit in any of the other groups | | | | | |
| **Year** | **Status Quo** | **HAART initiation according to Mexican guidelines** | **HAART initiation according to WHO guidelines** | **HAART initiation according to Mexican guidelines + 80% of diagnoses increased** | **HAART initiation according to WHO guidelines + 80% of diagnoses increased** |
| **1^st^ year** | 24,836 | 24,222 | 23,973 | 23,835 | 23,170 |
| **5^th^ year** | 147,607 | 135,871 | 129,919 | 98,428 | 85,602 |
| **10^th^ year** | 347,051 | 297,710 | 275,874 | 140,019 | 114,347 |
| **15^th^ year** | 605,450 | 484,985 | 436,357 | 156,810 | 124,126 |
| **20^th^ year** | 939,640 | 701,671 | 612,885 | 164,311 | 128,137 |

*Simulation of the model in the scenario Status Quo for the first definition of the sexual risk groups*

BERKELEY MADONNA CODE

METHOD RK4

STARTTIME = 0 ; months

STOPTIME = 240 ; months

DT = 1

{===================================================== }

{ Differential equations }

{===================================================== }

{HIV susceptible}

d/dt (S) = a*N - S* lambda_Inodx - S* lambda_Idx - S* lambda_Tnosup - S* lambda_Tsup - m * S ;

{HIV infectious without diagnoses}

d/dt(Inodx)= lambda_Inodx*S + lambda_Idx*S+lambda_Tnosup*S+lambda_Tsup*S - ro*Inodx - m*Inodx - mu*Inodx ; HIV without diagnoses

{HIV with diagnoses}

d/dt (Idx) = ro*Inodx - tao*Idx - mu * Idx - m *Idx ; HIV with diagnoses,

{HIV in treatment without suppression}

d/dt (Tnosup) = tao*Idx - sigma*Tnosup - mu* Tnosup - m *Tnosup ; HIV with diagnoses,

{HIV in treatment and suppressed}

d/dt(Tsup) = sigma*Tnosup - m*Tsup - mu*Tsup

; Annual number of deaths due to HIV in diagnosed people

d/dt(D) = mu *( Tsup + Tnosup + Idx)

{===================================================== }

{ Initial conditions and parameters }

{===================================================== }

INIT S = S_0 ; Initial number of HIV_Sus

INIT Inodx = Inodx_0 ; Initial number of HIV_Inf who is unaware of their status

INIT Idx = Idx_0 ; Initial number of HIV_Inf diagnosed

INIT Tnosup = Tnosup_0 ; Initial number of HIV_treated without suppression

INIT Tsup = Tsup_0 ; Initial number of HIV_treated with suppression

INIT D = D_0

S_0 = 1688600

N = 30960084 ;INEGI, 2011 ages 15 to 54 year

Inodx_0 = 114924 ; 68% without treatment are unaware of their status

Idx_0 = 9735

Tnosup_0 = 12861 ; 44347 on treatment, 71% with viral suppression

Tsup_0 = 31486

D_0 = 0

prop_H = 0.006 ; initial proportion of population in high activity group

prop_M = 0.064

prop_L = 0.93

c_H = 15.2 ; ptr change rate of high activity group / month

c_M = 2.5

c_L = 0.65 ; ptr change rate of low activity group / month

p = 0.73 ; proportion of sexual acts with protection

efic = 0.84 ; efficacy of condom

p_rec = 0.64 ; probability of an HIV- being receptive

rate_rec = 0.0143 ; probability of HIV transmission by being receptive

p_ins = 0.36 ; probability of being insertive

rate_ins = 0.0062 ; probability of HIV transmission by being insertive

numcontact_H = 1 ; number of sexual contacts per partnership in high risk (mean of 2.5 sex contacts per partnership)

numcontact_M = 1

numcontact_L = 2.87 ; number of sexual contacts per partnership in low risk

beta_a = (1- efic*p)*(p_rec*rate_rec + (1- p_rec)*rate_ins ) ; transmission probability per sexual act

beta_p_H = (1-beta_a)^(numcontact_H) ; No transmission probability per partner of high risk in all the sexual contacts

beta_p_M = (1-beta_a)^(numcontact_M)

beta_p_L = (1-beta_a)^(numcontact_L) ;No transmission probability per partner of low risk in all the sexual contacts

beta_p = (1 - ( beta_p_H^ c_H) )*prop_H + ( 1- ( beta_p_M^c_M) ) * prop_M + ( 1 - ( beta_p_L^c_L) ) *prop_L ; Transmission probability per all the partners of different sexual risk

ro = 0.015 ; 1/time since infection until diagnosis with a CD4 of 209

tao = 0.0544 ; 1/time between diagnosis to treatment with a CD4 of 148

m = 0.00029 ; background mortality rate / month. INEGI crude mortality rate 2011 in men of 15-55 yrs

a = 0.0411 ; recruitment rate / month (group median 17 yrs)

mu = 0.0027 ; death rate by AIDS /month. 3189 MSM died by AIDS in 2011, there was 97473 HIV+ MSM estimated

{===================================================== }

{ Useful calculations }

{===================================================== }

sigma = 0.041; rate of suppression per month. 71% of treated people reach suppression 6 mo later their HAART initiation and keeps their status during all their lifetime. Non suppressed includes people who suppressed and then interrupt their treatment.

lambda_Inodx = HIVprevInodx*(beta_p)

lambda_Idx = HIVprevIdx*(beta_p)

lambda_Tnosup = HIVprevTnosup*(beta_p)

lambda_Tsup = HIVprevTsup*0.00008 ; Attia, rate of 0.01/100 yrsperson for people on ART

{===================================================== }

{ Useful output }

{===================================================== }

; Numbers in groups

U= S+Inodx + Idx + Tnosup + Tsup ; number sex active in the high activity group

; Prevalences

HIVprevInodx = Inodx / U

HIVprevIdx = Idx / U

HIVprevTnosup = Tnosup /U

HIVprevTsup = Tsup /U

HIVprev = (Inodx+Idx+Tnosup + Tsup)/ U

; New HIV infections due to each group (Non diagnosed, diagnosed without tx, diag treated without suppression)

new_Inodx = lambda_Inodx * S

new_Idx = lambda_Idx* S

new_Tnosup = lambda_Tnosup *S

new_Tsup = lambda_Tsup*S

new_I = new_Inodx + new_Idx+ new_Tnosup + new_Tsup

***References***

1. Huang X, Lodi S, Fox Z, Li W, Phillips A, Porter K, et al. Rate of CD4 decline and HIV-RNA change following HIV seroconversion in men who have sex with men: a comparison between the Beijing PRIMO and CASCADE cohorts. Journal of acquired immune deficiency syndromes (1999). 2013 Apr 1;62(4):441-6.

2. Bautista-Arredondo S, Colchero MA, Romero M, Conde-Glez C, Sosa-Rubí S. Is the HIV epidemic stable among MSM in Mexico? HIV prevalence and risk behavior results from a nationally representative survey among men who have sex with men. PLoS ONE. 2013;8(9):e72616.

3. INEGI. Distribución por edad y sexo. Población total por grupo quinquenal de edad según sexo, 1950 a 2010. 2011 [cited 2012 10 23 ]; Available from: <http://www.inegi.org.mx/sistemas/sisept/Default.aspx?t=mdemo03&s=est&c=17500>.

4. Lieb S TD, Misra S, Gates GJ, Duffus WA, Fallon SJ, Liberti TM, Foust EM, Malow RM; Southern AIDS Coalition MSM Project Team Estimating populations of men who have sex with men in the southern United States. J Urban Health. 2009;Nov;86((6)):887-901.

5. INEGI. Mortalidad. Defunciones según sexo por entidad federativa 2010 a 2012. 2011.

6. CENSIDA. Vigilancia Epidemiológica de casos de VIH/SIDA en México. Registro Nacional de Casos de SIDA.Actualización al 30 de Junio del 2012. 2012 [updated 30/06/12; cited 2012 23/10]; Available from: <http://www.censida.salud.gob.mx/descargas/2012/sida_vih30junio2012pub2.pdf>.

7. CENSIDA. Informe Nacional de Avances en la Lucha contra el SIDA, México 2012. Mexico2012 [cited 2012 Aug 25]; Available from: <http://www.unaids.org/en/dataanalysis/knowyourresponse/countryprogressreports/2012countries/Informe%20Narrativo%202012_Mexico20120625.pdf>.

8. CENSIDA. Boletín Datos a 30 junio 2012, SALVAR Sistema de Administración Logística y Vigilancia ARV. México: Secretaria de Salud, 2012 06/30/2012. Report No.: 17.

9. CENSIDA. Boletín SALVAR N° 15. 2011 [cited 2013 Aug 23]; Available from: <http://www.censida.salud.gob.mx/descargas/atencion/Boletin_Salvar15.pdf>.

10. D'Anna LH, Margolis AD, Warner L, Korosteleva OA, O'Donnell L, Rietmeijer CA, et al. Condom use problems during anal sex among men who have sex with men (MSM): findings from the Safe in the City study. AIDS Care. 2012;24(8):1028-38.

11. Jin F Fau - Jansson J, Jansson J Fau - Law M, Law M Fau - Prestage GP, Prestage Gp Fau - Zablotska I, Zablotska I Fau - Imrie JCG, Imrie Jc Fau - Kippax SC, et al. Per-contact probability of HIV transmission in homosexual men in Sydney in the era of HAART. AIDS. 2010 20100317 DCOM- 20110325;24(1473-5571 (Electronic)):907–13.

12. Bravo-García E, Ortiz-Pérez H, editors. Eight years of free and universal access to HAART in Mexico: spending on treatment for HIV/AIDS grew more than 100%, but does not decline AIDS mortality. AIDS; 2012; Washington, DC.

13. INEGI. Distribución por edad y sexo. Población total por grupo quinquenal de edad según sexo, 1950 a 2010. 2011 [cited 2011 Feb 16]; Available from: <http://www3.inegi.org.mx/sistemas/sisept/Default.aspx?t=mdemo03&s=est&c=17500>.

14. UNAIDS. Epidemiology Fact Sheet on HIV and AIDS estimates. 2011 [cited 2013 Aug 23]; Available from: file:///C:/Users/Yanin/Documents/Doc/Doc/Parametros%20del%20modelo/Epidemiology%20fact%20sheet%20ONUSIDA_230813.htm.

15. Magis C, Bravo-García E, Uribe P. Dos decadas de la epidemia de SIDA en México. 2000 [cited 2013]; Available from: <http://www.censida.salud.gob.mx/descargas/dosdecadas.pdf>.

16. Factors associated with short-term changes in HIV viral load and CD4(+) cell count in antiretroviral-naive individuals. AIDS (London, England). 2014 Jun 1;28(9):1351-6.

17. http://www.chip.dk/portals/0/files/CROI_2014_PARTNER_QA.pdf

18. Attia S, Egger M, Muller M, Zwahlen M, Low N. Sexual transmission of HIV according to viral load and antiretroviral therapy: systematic review and meta-analysis. AIDS. 2009 Jul 17;23(11):1397-404.
